# Uncoupling hepatic insulin resistance – hepatic inflammation to improve insulin sensitivity and to prevent impaired metabolism-associated fatty liver disease in type 2 diabetes

**DOI:** 10.3389/fendo.2023.1193373

**Published:** 2023-06-15

**Authors:** Sitara Niranjan, Brett E. Phillips, Nick Giannoukakis

**Affiliations:** ^1^ Department of Internal Medicine, Allegheny Health Network, Pittsburgh, PA, United States; ^2^ Department of Biological Sciences, Carnegie Mellon University, Pittsburgh, PA, United States

**Keywords:** insulin resistance, type 2 diabetes, NAFLD, NASH, hepatic insulin resistance

## Abstract

Diabetes mellitus is a metabolic disease clinically-characterized as acute and chronic hyperglycemia. It is emerging as one of the common conditions associated with incident liver disease in the US. The mechanism by which diabetes drives liver disease has become an intense topic of discussion and a highly sought-after therapeutic target. Insulin resistance (IR) appears early in the progression of type 2 diabetes (T2D), particularly in obese individuals. One of the co-morbid conditions of obesity-associated diabetes that is on the rise globally is referred to as non-alcoholic fatty liver disease (NAFLD). IR is one of a number of known and suspected mechanism that underlie the progression of NAFLD which concurrently exhibits hepatic inflammation, particularly enriched in cells of the innate arm of the immune system. In this review we focus on the known mechanisms that are suspected to play a role in the cause-effect relationship between hepatic IR and hepatic inflammation and its role in the progression of T2D-associated NAFLD. Uncoupling hepatic IR/hepatic inflammation may break an intra-hepatic vicious cycle, facilitating the attenuation or prevention of NAFLD with a concurrent restoration of physiologic glycemic control. As part of this review, we therefore also assess the potential of a number of existing and emerging therapeutic interventions that can target both conditions simultaneously as treatment options to break this cycle.

## Introduction

Diabetes mellitus is a metabolic disease clinically-characterized as acute and chronic hyperglycemia (1). It is emerging as one of the common conditions associated with incident liver disease in the US. The spectrum of liver disease ranges from mild transaminitis to non-alcoholic fatty liver disease (NAFLD). NAFLD encompasses non-alcoholic steatosis (fatty liver) without inflammation (normal transaminases), non-alcoholic steatohepatitis (NASH) without fibrosis, NASH with fibrosis eventually progressing to cirrhosis, hepatocellular carcinoma and liver failure culminating in death (1, 2). In clinical practice, most patients with NAFLD are asymptomatic with possible hepatomegaly. They are diagnosed when liver enzymes ALT and/or AST are elevated, or steatosis is detected on abdominal imaging. It is a diagnosis of exclusion, and normal liver enzymes do not eliminate a diagnosis of NAFLD (3–7). Worldwide, the pooled prevalence of NAFLD (umbrella term of macrovesicular fat deposition) is 25.24% (8). In the US, a comparison of 3 cycles of the National Health and Nutrition Examination Survey (NHANES) based on transaminitis alone, demonstrated a steady increase in the prevalence of NAFLD from 5.5% in 1988 to 11% in 2008. The inclusion of steatosis with normal transaminases may account for an even higher prevalence (9). The prevalence of NAFLD’s closely associated metabolic counterparts such as essential hypertension, obesity and diabetes has trended up as well (10). Studies in multiple countries have demonstrated that NAFLD has a higher prevalence in men. Prevalence in women increases with age, while it remains stable in men. Sex hormones, menopausal status and obesity are major contributing factors to this disparity (11).

The mechanisms by which diabetes drives liver disease have become a topic of intense discussion and highly sought-after therapeutic targets. Traditionally, diabetes has been classified into type 1 (T1D) and type 2 (T2D). T1D begins as an autoimmune process culminating in an autoimmune inflammation-mediated, selective impairment of the pancreatic beta cells and overt hyperglycemia. T2D, instead, is characterized by peripheral insulin resistance (IR) compensated for by the production of more insulin culminating in overt hyperglycemia. Accumulating evidence suggests that these seemingly divergent conditions share many etiopathogenetic and clinical features other than just hyperglycemia. Thus, latent autoimmune diabetes of adults (LADA) presents features of both T1D and T2D and IR is seen in overweight T1D patients (12). On the other hand, some T2D patients exhibit pancreatic autoimmunity (13).

## Evolution of hepatic IR in T2D and T2D-associated NAFLD

**Figure 1:**
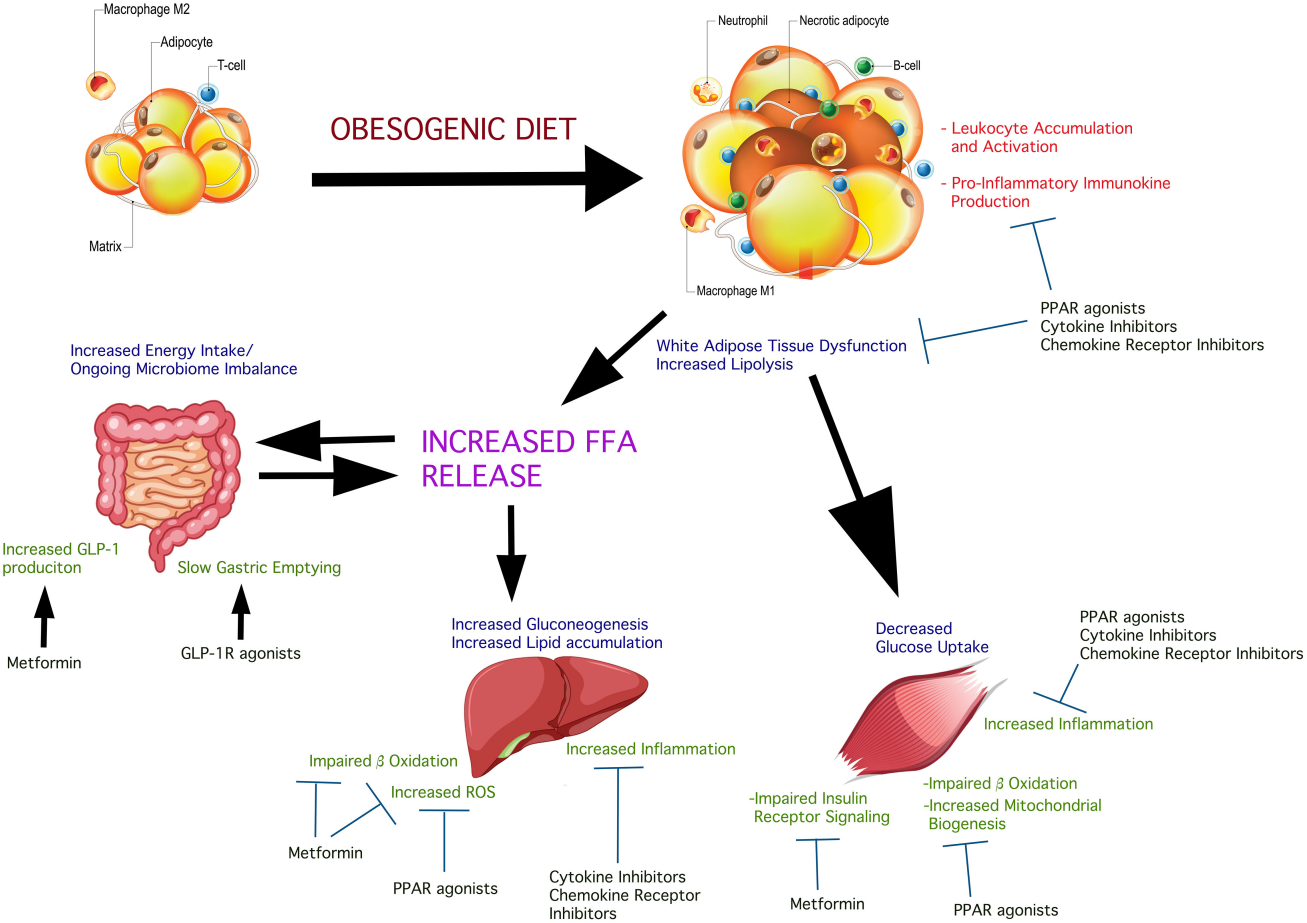
Insulin-sensitive inter-organ effects of obesogenic diets in the deterioration of insulin sensitivity and possible treatment pressure points. Obesogenic diets promote a state of systemic low-grade inflammation which contributes to, and is response to pathologic changes in glucolipometabolism in the main insulin-sensitive tissues and organs. The intestinal microbiome is altered causing changes in the complement of short-chain fatty acids produced. These are released into the circulation affecting insulin sensitivity and potentiate systemic and insulin-sensitive tissue inflammation. Obesogenic diet causes adipose hypertrophy and expansion, resulting in the conversion of resident M2 macrophages into pro-inflammatory M1 macrophage. Concurrent core adipose hypoxia creates an environment that signals “danger”. This initiates the accumulation of neutrophils and other leukocytes which become activated, further potentiating local inflammation. The net result is peripheral insulin resistance, consequent to insulin receptor signaling impairment due to the action of immunokines produced by the accumulating pro-inflammatory leukocytes. IL-6, for example, acting *via* the IL-6 receptor on adipocytes, impairs insulin-stimulated phosphorylation of signaling components downstream of the insulin receptor tyrosine kinase. Skeletal muscle is susceptible to expanding fat and accumulation of lipid droplets, as well as the effects of circulating FFA on hepatocytes cause the accumulation of leukocytes and their activation, with a net effect of insulin signaling impairment. The different classes of agents shown in the Figure have shown variable beneficial effects on insulin sensitivity. Combination approaches could simultaneously act systemically and on the key insulin-sensitive tissues, attenuating inflammation thus facilitating better insulin sensitivity.

Broadly-understood, IR is coupled to impaired insulin action at multiple points in the signaling cascade in the main glucose-utilizing, insulin-responsive tissues, particularly skeletal muscle, adipose, and the liver. These as well as possible pressure points of therapeutic interest are illustrated in **Figure 1**. These include the action of lipid mediators, cellular stress, mitochondrial abnormalities, and leukocyte-derived soluble molecules (14). Lipid-induced IR has been observed in the liver (15) as the consequence of high fat diet (HFD) or lipolysis, where the concentration of FFA exceeds that of the intracellular fatty acid oxidation and storage rate, as demonstrated in humans and rodent models (14). Increased concentrations of diacylglycerol (DAG) also lead to IR by impairing insulin signaling (14). For example, plasmalemmal accumulation of intrahepatic DAG stimulates protein kinase Cϵ and inhibitory insulin receptor kinase phosphorylation on threonine (16, 17) resulting in IR. These results were consistent in rodent models and humans. In addition to protein kinase Cϵ, increased activity of the δ enzymatic isoform in livers of obese humans has been observed to cause hepatic IR (18). Human study outcomes and rodent models have shown that activation of other protein kinase C isoforms (δ, ϵ, ν, θ) have been implicated in DAG release and IR onset or progression (14). Non-FFA-derived lipids are another species implicated in the onset of hepatic IR in humans exhibiting NASH. A number of studies in humans revealed elevated intra-hepatic FFA concentrations concurrent with hepatic oxidative stress and inflammation (19). While ceramides have also been implicated in hepatic IR under obese conditions and T2D evolution, this has been well-reviewed elsewhere (20) and remains outside the topic of the current review. While HFD-facilitated elevations in circulating FFAs and lipids as a basis of IR is strongly-supported by many lines of animal and human investigation (21), not all situations of IR are a consequence of this. Cellular stress, instead, is a better predictor of IR in the obese state. Endoplasmic reticulum (ER) stress, particularly, is a common finding in the liver among obese men and women (22, 23). Nevertheless, exposure to HFD in rodents leads to an expansion of lipid deposition inside the liver followed by hepatic IR even in the absence of peripheral fat accumulation and peripheral IR. Under such diet conditions, insulin signaling has been shown to be impaired, partly due to activation of PKCε and JNK1 (24). Estrogen has a protective role against hepatic steatosis and insulin resistance by decreasing triglyceride synthesis and increasing hepatic FFA oxidation (25). Circulating 17-beta estradiol also suppresses hepatic gluconeogenesis *via* FoxO1 signaling, independent of IRS-1 and IRS- 2 (26). In mice IRS-2 is transcriptionally-attenuated as a function of sterol-regulatory element binding protein (SREBP) activation and FoxO suppression (27–31). This is possibly a consequence of hyperinsulinemia-induced downregulation of IRS-2 facilitating hepatic IR (32, 33). Further, growing evidence indicates that hepatic DAG accumulation potentiates hepatic IR (34) and DAG levels inside hepatocyte lipid droplets were particularly-informative predictors of IR in humans (35).

## The paradox of increased hepatic lipogenesis in the presence of hepatic IR

One of the molecular pathways of insulin signaling is the activation of Akt which, as it suppresses hepatic gluconeogenesis, in parallel causes activation of sterol regulatory element binding protein 1c (SREBP1c). As demonstrated in transgenic rat hepatocytes, this is a consequence of Akt-stimulated mammalian target of rapamycin complex-1 (mTORC1) activity which regulates the transcription and stability of SREBP1c (36). Activated SREBP1c stimulates increased expression of genes encoding key enzymes in FA biosynthesis including those of the fatty acid elongase complex, fatty acid synthase (FAS), acetyl-CoA carboxylase (ACC), and ATP citrate lyase (37). A seeming paradox is observed inside the liver with developing obesity and progression towards T2D-associated NAFLD. Impairment of gluconeogenesis suppression occurs concomitant with *de novo* lipogenesis (DNL) and IR. This can be partially explained as a function of liver insulin signaling stimulating hepatic DNL whose biochemical pathway products predispose and drive the impairment of gluconeogenesis suppression. These biochemical pathway products and their concentrations, evidence suggests, determine the onset and rate of hepatic structural and cellular damage observed in the onset of NAFLD in mice (38). The question that remains to be better understood is, what is the point in hepatic insulin signaling where its effects on glucose and lipid metabolism diverge?

Some evidence suggests that mTORC1 may be one such point of divergence at the level of hepatic hyperinsulinemia and resistance. Studies in rodents have shown that the blockade of Akt and PI3K activity prevents insulin-mediated expression of genes of enzymes involved in gluconeogenesis while mTORC1 prevented insulin-dependent induction of SREBP1c without any effect on suppression of expression of gluconeogenetic genes (39). mTORC1 is a nutrient-sensing biochemical control point promoting its re-distribution to the lysosome (40–42). However, as demonstrated in transgenic mouse models, mTORC1, on its own, is insufficient to cause *de novo* lipogenesis and NASH, at least in the absence of Akt2 (43). The nuance in these observations is best evaluated noting the role of tuberous sclerosis complex (TSC) proteins (44, 45). A number of mouse models have shown that Akt stimulation of mTORC1 is conditioned on TSC2 inhibition. Hepatic deletion of TSC1 results in an insulin-depended mTORC1 activation and protects from steatosis and *de novo* lipogenesis (44, 46). Additional studies in mice exhibiting hepatocyte-targeted inactivating genetic modifications of Akt, FoxO1, and TSC1, insulin-dependent co-ordinate activation of mTORC1 and FoxO1 inhibition were considered to be sufficient and possibly-necessary for insulin-dependent *de novo* lipogenesis (47, 48).

## The stress response as one of the mechanisms involved in the evolution of hepatic IR

Co-incident with the onset of NAFLD, are a series of changes inside hepatocytes indicating an acute stress response; changes concomitant with intra-hepatic inflammation (49). Central to this stress response is the unfolded protein response (UPR) with its fulcrum point the endoplasmic reticulum (ER). Hepatic ER stress has been observed in NAFLD (50) and related to its progression, including its mechanistic relationship with hepatic insulin resistance (51). ER stress has been coupled to steatohepatitis-associated insulin resistance (52). Moreover, *de novo* lipogenesis in the liver has also been linked to hepatocyte ER stress (53). Pharmacologic suppression of Caspase-2 as well as Caspase-2 disruption, observed in hepatocyte ER stress-associated NASH prevented fibrosis and inflammation by preventing SREBP1 and SREBP2 activation. These observations suggested that ER stress could participate in the early onset of hepatic insulin resistance, *de novo* lipogenesis and the progression towards NAFLD.

## Amino acids in the evolution of hepatic IR

It stands to reason that, especially under HFD conditions, lipids and FFAs are widely-viewed as the basis of IR, systemic or hepatic, however, other metabolites, especially in high fat “Western diets” have been implicated. Several amino acids (AA) have been shown to contribute to IR (15). In humans, AA elevation in plasma impairs insulin-stimulated glucose disposal in skeletal muscle. The mechanism appears to be through the mammalian target of rapamycin (mTOR)/S6 kinase pathway and phosphorylation of IRS-1 (54). Branched-chain (BC) AA are constituents of liver gluconeogenesis and their levels in the circulation have been found to be correlated with IR in humans (55). In skeletal muscle under hypersinulinemic conditions, BCAA impair glucose disposal and augment ATP synthesis without any effect on mitochondrial abundance of DNA (56, 57). In contrast, transient dietary reduction of BCAA reduces post-prandial insulin secretion and improves adipose metabolism (58).

## Leukocytes, immunokines, and inflammation: cause or outcome of hepatic IR, in response to metabolic stress?

Macrophages are possibly the first leukocytes to accumulate inside the liver of obese individuals concomitant to IR onset (peripheral and/or hepatic) (59). These cells impair insulin signaling mainly *via* secreted immunokines (60). Liver-resident macrophages have been implicated in the onset and progression of hepatic IR and a number of overlapping mechanisms have been identified in their activation. While the following observations have been made mainly in skeletal muscle, and muscle-associated adipose, one can anticipate similar mechanisms to participate in hepatic IR: Accumulation of lipids inside myotubes in humans and rodent models, stimulates NF-κB nuclear translocation, attenuated mitochondrial respiration, fragmentation and mitophagy and elevated production of reactive oxygen species (ROS) (61). Systemic IR is widely-reported to co-incide with macrophage accumulation and activation inside adipose (62), however, adipose IR can manifest adipose macrophage accumulation and activation (63), suggesting that, at least in some instances, IR can precede an inflammatory state and may in fact represent a “danger” signal causing the eventual activation of Kupffer cells and liver macrophages. Potential mechanisms underlying an IR-first cause could involve local hyperinsulinemia-stimulated activation of these leukocytes and/or hyperinsulinemia-stimulated increase in microvascular blood flow, hyperoxygenation and hepatic cell stress. Hyperinsulinemia would then be a consequence of pancreatic β cell impairment. A number of known mechanisms of peripheral IR could cause beta cell impairment *via* stress induction, UPR, and failure to sense glucose/produce insulin (64, 65).

Overnutrition and obesity lead to a systemic low grade chronic inflammatory state referred to as meta-inflammation, characterized by adipocyte necrosis and altered secretory phenotype in adipocytes (66–68). This results in the recruitment and release of proinflammatory cells and cytokines, such as TNFα expressed by macrophages and monocytes infiltrating obese adipocytes. Adipose tissue contains predominantly M2 macrophages, with a phenotypic switch to M1 in obese persons. M1 macrophages produce chemokines such as MCP-1 which recruit circulating monocytes to the liver and adipose tissue where they can undergo maturation into the pro-inflammatory M1 phenotype. Adipocytes also produce low levels of TNFα, leading to MCP-1 production and macrophage infiltration in adipocytes, triggering release of pro-inflammatory cytokines, such as IL-6 and IL-1β (69). The level of pro inflammatory cytokines in subcutaneous abdominal adipose tissue, inversely correlates with hepatic and systemic insulin sensitivity. Obese individuals with NAFLD have shown a decrease in hepatocyte insulin signaling compared to obese individuals with normal intrahepatic triglycerides (70). This low grade chronic inflammatory state in adipose tissue further contributes to IR *via* TNFα mediated serine/threonine phosphorylation of IRS-1, leading to enhanced lipolysis and increased exposure of hepatocytes to lipids (71, 72), fueling the progression of NASH.

More recent human and rodent studies, however, show that macrophages alone may not be sufficient to be involved in hepatic pathology concomitant to obesity-driven IR. Accumulation of neutrophils occurs very close to, or concurrent with that of macrophages (73). Indeed, more recent data demonstrate a prominent role of neutrophils over macrophages as being pivotal leukocytes that license and co-operate with macrophages in the onset of IR and T2D (74, 75). Neutrophil migration to sites of “danger” and their activation is a function of the balance of the CXCR2/CXCR4 chemokine receptor density on their surface (76). Neutrophil-attracting CXCR2 ligands are expressed in the pancreas, adipose and liver (77), suggesting that under potentially-stressful states, their secretion can be expected to recruit and activate neutrophils, which in turn would exacerbate and amplify a low grade inflammatory condition (78).

With the activation of leukocytes inside the liver, such as macrophages, growing intra-hepatic lipid deposition results in immunokine release [reviewed in (79) and (80)] which potentiates adipocyte lipolysis (81) concomitant to inhibition of hepatic insulin signaling (81, 82). Immunokines promote not only hepatic, but also systemic IR (83, 84), and cytokines like TNFα are detectable and upregulated in concentration inside the liver and adipose tissue of NASH patients (85), suggesting that upregulated TNFα in adipose might potentiate the progression of NAFLD in two ways: systemic IR and activation of a peripheral inflammation of insulin-responsive tissues (86). For example, adipose-produced IL-6 in liver stimulates hepatic SOCS3, suppressing insulin signaling, resulting in hepatic IR (87). Serum IL-6 concentrations are elevated in NAFLD and NASH (88).

## Possible strategies to improve hepatic insulin sensitivity

The most obvious approaches to improving insulin sensitivity are diet changes and exercise that result in weight loss. However, work-life balance, in many instances, can impede commitment to defined diet and even low-level exercise activity. The distinct sex related disparities in the prevalence of NAFLD due to an interplay of sex hormones, age related hormonal changes as well as diseases such as polycystic ovarian syndrome and Turner’s Syndrome may warrant exploration into sex-specific therapeutic strategies that have been presented and/or reviewed elsewhere (89–93).

An array of different medicinals has been developed specifically to lower glucose concentrations, improve insulin production and/or correct weight and attenuate inflammation. **Table 1** presents the clinical studies where insulin sensitivity, and hepatic insulin sensitivity in particular, was one of the outcome measures. Other classes of drugs have been repurposed for these indications. Their effects on IR have been mild to variable. A single class of agent to improve insulin sensitivity together with prevention of IR-associated liver pathology remains to be discovered, although we have shown that a neutrophil-targeting CXCR2 antagonist could offer such a solution [see below, (105)].

**Table 1:** Clinical trials assessing the 3-month (and greater) outcomes on insulin sensitivity in overweight/obese individuals with or without type 2 diabetes.

Study Agent	Study Design	Main Outcome(s)	Metabolic Outcomes	Reference
Lixisenatide vs Placebo	Randomized, Placebo-controlled	Decreased HbA1c	Decreased - FPG - BW - 2hr PPG Increased - HOMAβ	Ahren et al. (94)
Dulaglutide vs Liraglutide	Randomized, Parallel	Decreased HbA1c	Decreased - FPG - BW - PPG	Dungan et al. (95)
Exenatide vs Placebo	Randomized, Placebo-controlled	Decreased - HbA1c - Hepatic triglycerides - Epicardial adipose	Decreased - BW	Dutour et al. (96)
Dulaglutide vs Liraglutide vs Placebo	Randomized, Placebo-controlled	Decreased HbA1c	Decreased (both agents vs. placebo) - HbA1c - FPG Increased - HOMA-2 %β	Miyagawa et al. (97)
Empagliflozin vs Placebo	Randomized, Placebo-controlled	Decreased Hepatic Lipid Content	Decreased - FPG - BW - Uric acid	Kahl et al. (98)
SAR425899 vs Liraglutide vs Placebo	Randomized, Parallel	Decreased HbA1c (both agents vs. placebo) Increased - HOMA-2 %S		Schiavon et al. (99)
Saroglitazar vs Placebo	Randomized, Placebo-controlled	Increased - Glucose Metabolism (M) - Insulin Sensitivty (M/I) - HOMA-β	Decreased - HbA1c - FPG - Triglycerides Increased - HDL-C	Jain et al. (100)
Pioglitazone vs Placebo	Randomized, Placebo-controlled	Increased - Glucose Disposal Rate - Insulin-Stimulated Suppression of Endogenous Glucose Production	Decreased - HbA1c - FPG - Plasma TG - Visceral Fat - BW Increased - BW - Fat Mass - Subcutaneous Fat	Miyazaki et al. (101)
Semaglutide vs Empagliflozin	Randomized Active Control	Decreased - HbA1c	Decreased - FPG - Fasting Plasma Insulin - Fasting C-Peptide - BW - CRP	Rodbard et al. (102)
Canagliflozin vs Placebo		Decreased - Hepatic Triglycerides Increased - Insulin-Stimulated Suppression of Endogenous Glucose Production - Beta Cell Function	Decreased - HbA1c - FPG - Fasting Plasma Insulin - BW Increased - Insulin Clearance - FFA	Cusi et al. (103)
Saroglitazar vs Pioglitazone	Randomized, Parallel	Decreased - HbA1c - FPG	Decreased - Triglycerides - VLDL-C - LDL-C - HDL-C	Krishnappa et al. (104)

## Antihyperglycemic agents

Sulfonylureas lower blood sugar concentrations by stimulating insulin secretion independent of food intake, however, they are associated with hypoglycemia. While some studies demonstrated beneficial effects on IR, others could not (106, 107). Sulfonylurea use is slowly being replaced by newer agent classes to treat hyperglycemia.

Metformin remains a first-line glucose lowering agent. Although the underlying mechanism of action remains incompletely understood, it appears that it inhibits the hepatic glycerol-3-phosphate dehydrogenase activity, resulting in suppression of glycerol-induced gluconeogenesis and increased cytosolic redox state. Together, these actions lead to a reduction in lactate dehydrogenase and lactate-induced endogenous glucose production (108). Other possible mechanisms of action include the inhibition of complex I followed by increased AMP, activating AMP kinase and facilitating fatty acid oxidation in liver and reduced expression of genes encoding enzymes involved in gluconeogenesis. Additionally, AMP interferes with glucagon signaling and gluconeogenesis (108). In non-hepatic tissues, metformin increases insulin stimulated glucose utilization (108) and AMP kinase activity (109). A meta-analytic inspection of 11 randomized controlled trials (RCT) in obese and overweight adolescents, revealed that metformin reduced fasting plasma glucose (FPG) at less than 6 months, without impacting insulin sensitivity (110). Another meta-analysis of 31 RCT using metformin for more than 8 weeks in individuals at high risk for T2D revealed that it improved insulin sensitivity concurrent with a reduced incidence T2D (111). An additional meta-analysis in patients with NAFLD revealed benefit in insulin sensitivity without, however, any improvement in NAFLD liver histology (112).

## Peroxisome proliferator-activated receptor agonists

PPAR agonists, particularly those for PPARγ, have shown promising efficacy in improving IR and liver histology in T2D-associated NAFLD. As a class, they also suppress the production of pro-inflammatory immunokines concurrent with stimulation of adiponectin production (113, 114). Pioglitazone treatment of T2D patients has resulted in beneficial outcomes in NAFLD (62) resulting in improved liver and peripheral insulin sensitivity (101). While its use has been somewhat questioned due to adverse event concerns (115), a more recently-developed agent, lobeglitazone, exhibits improved safety with improvements in insulin sensitivity and liver steatosis in T2D-associated NAFLD (116). Another PPARγ-sparing agent, MSDC-0602K, also achieves insulin-sensitizing peripheral effects safely (117). More recently, CHS-131 demonstrated significant dual-target outcomes, improving fasting insulin levels and insulin sensitivity, total plasma cholesterol, triglycerides, liver enzymes, and increased plasma adiponectin levels. Most importantly, CHS‐131 improved liver histology and markers of hepatic fibrosis (118). Fibrates, ligands of PPARα, reduce fasting plasma glucose, insulin, and improve insulin sensitivity (119) although some questions remain about their true efficacy (120). Seladelpar and GW501516 are PPARδ agonists shown to improve insulin sensitivity in obese individuals (120, 121) with mechanisms of action that include increased fatty acid oxidation in skeletal muscle and attenuation of macrophage pro-inflammatory state (122). Another PPAR agent is Elafibranor, a PPARα/δ agonist, which reduces inflammation and enhances both peripheral and liver insulin sensitivity under obese conditions (123, 124), although the latter findings remain to be validated (125). Saroglitazar is a dual PPARα/γ agonist with whole body insulin sensitivity improvement without adverse events noted with the use of other PPARα/γ agonists (100, 104). A pan-PPAR agonist, lanifibranor, is currently being tested in phase II studies, with enabling data showing improved insulin sensitivity in T2D and improved intra-hepatic lipid content in T2D-associated NAFLD (clinicaltrials.gov #NCT03459079).

## Fatty acid synthetases

A randomized single blinded phase 2a clinical trial evaluated the efficacy of a fatty acid synthetase inhibitor TVB-2640 on *de novo* lipogenesis in a population of NASH patients (126). Fatty acid synthetases convert metabolites of simple sugars to palmitate (126). The rationale behind this was to reduce *de novo* lipogenesis in patients with NASH. The outcome demonstrated decreased liver fat by 9.6% in a population with fatty liver and fibrosis that included subjects with diabetes.

## Incretins

GLP-1 agonists like exenatide, liraglutide, semaglutide, and lixisenide can improve insulin sensitivity, although it is not clear if this effect is in the periphery or in the liver as well (94, 96, 102, 127, 128). Glucose-dependent insulinotropic polypeptide (GIP; tirzepatide) use also achieved some insulin sensitivity improvement in T2D, although again it is unknown if this acted at the level of the liver (127). Reduced hepatic inflammation and lipid deposition was demonstrated in T2D-associated liver pathology following a tri-pathway-targeting approach using HM1521, an agent that targets glucagon/GIP/GLP-1Ra in mice and in humans (117, 127).

## α-Glucosidase inhibitors and sodium glucose co-transporter-inhibitors

While α-Glucosidase inhibitors (AGI) are not *a priori* thought of as agents that could affect IR, clinical studies have shown that they can, following establishment of a steady dose level (129, 130). These effects are expected to be extra-hepatic and a consequence of attenuation of hyperglycemia. In a similar manner, Sodium Glucose Co-transporter-2 Inhibitors (SGLT2I) have also demonstrated some insulin sensitivity enhancing effect (103, 131, 132) including a positive effect on liver IR (103) with neutral outcomes on non-hepatic IR (133).

## Leukocyte and immunokine-targeting anti-inflammatory agents

It stands to reason that the accumulation of pro-inflammatory leukocytes and elevation of the concentration of their pro-inflammatory soluble mediators inside insulin-sensitive tissues is a high-priority target of therapy aimed to restore normal insulin-sensitivity in T2D as well as prevent any T2D-associated liver impairment that can be a consequence of, or drive hepatic IR. Salicylates were among the earliest agents tested for this objective and demonstrated mild improvement in peripheral glucose disposal (134, 135).

Inhibition of TNFα action with a variety of antibodies (etanercept, infliximab, adalimumab) improved insulin sensitivity in some patients, however, the heterogeneity of the study populations requires validation of those outcomes (135, 136). Targeting the IL-1β system (using IL-1 receptor antagonist protein, or antibodies like canakinumab and gevokizumab) improves glucoregulation overall, absent of any discernible effects on insulin resistance in T2D (135). In contrast, using the IL-6-targeting antibody tocilizumab, which aims to break the IL-6-mediated interference of insulin signaling, achieved statistically-relevant improvement of insulin sensitivity in obese patients (137).

Some excitement was generated when initial results from pre-clinical and early-clinical outcomes were reported showing improved hepatic function with the use of cenicriviroc, a dual CCR2/CCR5 chemokine receptor antagonist in hepatic pathology, however these reactions were tempered when the agent was unable to improve insulin sensitivity in patients with NASH (138).

As neutrophil accumulation into areas characterized by molecular and microenvironmental structural anomaly is a function mainly of the balance of CXCR2 and CXCR4 ligands and the neutrophil cell surface ratio of CXCR2:CXCR4 chemokine receptors (76), modulation of signaling *via* these receptors was proposed to be potentially therapeutic for T2D progression, IR, and possibly NAFLD. CXCR2-deficient mice are resistant from high fat diet-induced IR and T2D and are characterized by reduced macrophage accumulation in adipose (139). We recently demonstrated that a selective CXCR2 antagonist, AZD5069 (140) treatment of high fat diet-fed mice, improved insulin sensitivity and insulin-induced suppression of hepatic glucose production, decreased hepatic lipid storage, and significantly-prevented the progression towards liver pathology reminiscent of NAFLD.

Myeloperoxidase (MPO) is a key enzyme in neutrophil respiratory burst, that generates reactive oxidation species. Studies have shown an increase in the prevalence of MPO-positive Kupffer cells and neutrophils in the liver during NASH. The free radicals produced by MPO could participate in liver damage, directly (on hepatocytes) and/or on the stroma. MPO-deficient mice fed a high fat diet were protected against NASH-related liver injury. Additionally, mice fed with an oral MPO inhibitor exhibited reduced transaminitis and fibrosis (141). Thus, this enzyme, targeted alone or together with CXCR2 inhibitors/antagonists could represent a novel therapeutic approach in liver IR-related NASH (142, 143).

Currently there are no FDA-approved single agent treatments for the concurrent management of insulin sensitivity and the prevention (or at least the attenuation of progression to) to NAFLD/NASH in individuals with metabolic syndrome and T2D. The closest drug to market is obeticholic acid which recently completed a phase 3 clinical trial, but has yet to be approved by the FDA due to safety concerns in long term adverse effects (144). Our outcomes with AZD5069, as a single agent, showing benefits in the prevention of progression of insulin resistance and liver pathology reminiscent of NASH/NAFLD, as well as clinical trials in humans showing that AZD5069 was very well-tolerated with few side effects (145), offer an opportunity for this and possibly other similar drugs (e.g. ladarixin (146),) to enter clinical consideration as adjunctive treatments to standard of care of obesity and T2D to prevent and/or attenuate insulin resistance and liver pathology.

AZD5069 and similar agents may be found to exert their overall effects in a wider-ranging manner. For example, by preventing CXCR2-stimulated inhibition of insulin-induced glucose transport in muscle cells (147). Additionally, by preventing the effects of IL-8 (produced by growing adipose) on insulin-induced Akt phosphorylation in adipocytes (148, 149). This furthers strengthens the rationale that these agents can be potentially helpful treatments in insulin resistance-incident obesity and T2D. Finally, ongoing studies in our laboratory will soon determine if neutrophil antagonism impacts macrophage accumulation and function and thus, in an indirect manner, AZD5069 and similar agents, such as ladarixin (146), could prevent accumulation and further activation of liver-resident macrophages.

## Modulation of lipid and energy metabolism

Improvement in insulin sensitivity in obesity and T2D-associated NAFLD have been achieved using lipid metabolism-modifying agents like ketohexokinase inhibitor, a protein tyrosine phosphatase-1B inhibitor, or an ω3-fatty acid [reviewed in (117)]. Liver-targeted dinitriophenyl (DNP)-methyl ether (DNPME) and mitochondrial protonophore (CRMP) aiming to motivate hepatic fatty acid oxidation while reducing lipid accumulation improved systemic IR in rodent and non-human primate models of obesity-associated NAFLD (150). Another mitochondrion-acting agent, BAM15, also showed evidence of improving systemic IR and liver inflammation as well as pathology in mouse models of obesity (150). Precise targeting of sensitive points inside these pathways without systemic adverse events or toxicities remains a largely-unexplored area of T2D pharmaceutical research, especially for the objective of improving IR concurrent with delaying or obviating liver pathology.

## Discussion

It is now evident that inflammation dependent pathways have a clear pathological role in the propagation of NAFLD. Initially, IR and hepatic lipid accumulation result in oxidative stress and activation of inflammatory pathways in the liver. In fact, inflammation plays a key role in IR as well. Overnutrition and increased caloric intake, set the stage for IR *via* multiple mechanisms. IR and hepatic lipid accumulation result in oxidative stress and activation of inflammatory pathways in the liver. Additionally, ER stress culminates in the UPR aimed at reducing ER burden while simultaneously increasing the translation of pro-apoptotic proteins. Finally, obesity-mediated adipocyte inflammation and necrosis results in a systemic meta-inflammation mediated by macrophages and cytokines such as TNFα and IL-8. IR contributes to hepatic steatosis through an increase in the circulating FFA, further leading to inflammation dependent liver injury resulting in NASH. This happens through liver macrophages in combination with, as emerging evidence indicates, the increased recruitment of neutrophils through CXCR2 signals. This recruitment of inflammatory cells to the liver plays a key role in the pathogenesis of NASH. Functionally, peripheral IR, especially in the liver further impairs systemic glucoregulation. The liver is a key site of gluconeogenesis, typically down regulated by insulin *via* the interference in transcription of gluconeogenic genes. Insulin physiologically favors lipogenesis and inhibits gluconeogenesis. Paradoxically, during IR states in the liver, there continues to be an increase in lipogenesis and gluconeogenesis referred to as selective IR. This culminates in NASH and systemic hyperglycemia, contributing to the diabetic phenotype.

With respect to therapeutics, a novel approach is to target IR and interfere with the natural disease progression of NASH. Bearing in mind that IR often precedes NASH and has an overlapping pathogenesis in the form of systemic meta-inflammation, combination therapy targeting at least two distinct inflammation networks would have maximum synergistic value. CXCR2 antagonists are a novel approach that have demonstrated both an improvement in insulin sensitivity and interference in the natural disease progression of NASH, through an interference in recruitment of inflammatory cells. CXCR2 antagonists in combination with PPARγ agonists may have a synergistic role considering the latter’s proven efficacy in improving insulin sensitivity and potential in NASH treatment. PPARγ agonists improve insulin sensitivity by increasing adiponectin and GLUT-4 translocation. Though limited by their side effects such as pulmonary edema in clinical practices new alternatives like CHS-131 show promise in this aspect, alone or in combination.

## Author contributions

SN and BP wrote the original draft of the manuscript with significant intellectual guidance by NG. SN and NG edited all versions of the manuscript. NG assumes responsibility of the final submitted draft. All authors contributed to the article and approved the submitted version.
